# Downscaling of national crop area statistics using drivers of cropland productivity measured at fine resolutions

**DOI:** 10.1371/journal.pone.0205152

**Published:** 2018-10-11

**Authors:** Jingyu Song, Michael S. Delgado, Paul V. Preckel, Nelson B. Villoria

**Affiliations:** 1 Nationwide Mutual Insurance Company, Columbus, OH, United States of America; 2 Department of Agricultural Economics, Purdue University, West Lafayette, IN, United States of America; 3 Department of Agricultural Economics, Kansas State University, Manhattan, KS, United States of America; Michigan State University, UNITED STATES

## Abstract

Despite substantial research and policy interest in pixel level cropland allocation data, few sources are available that span a large geographic area. The data used for much of this research are derived from complex modeling techniques that may include model simulation and other data processing. We develop a transparent econometric framework that uses pixel level biophysical measurements and aggregate cropland statistics to develop pixel level cropland allocation predictions. Such pixel level land use data can be used to investigate the impact of human activities on the environment. Validation exercises show that our approach is effective at downscaling cropland allocation to multiple levels of resolution.

## Introduction

Agricultural productivity and environmental sustainability are central focuses for policymakers and academics. Understanding the interaction between agricultural systems and economic and environmental systems is critical for enhancing public policies related to economic development, food security, and human well-being. One important element to understand such interactions is the distribution of cropland and crop types within a particular area, particularly when the goal is to design policies that focus on promoting agricultural productivity while ensuring environmental sustainability [1–3].

**Fig 1:**
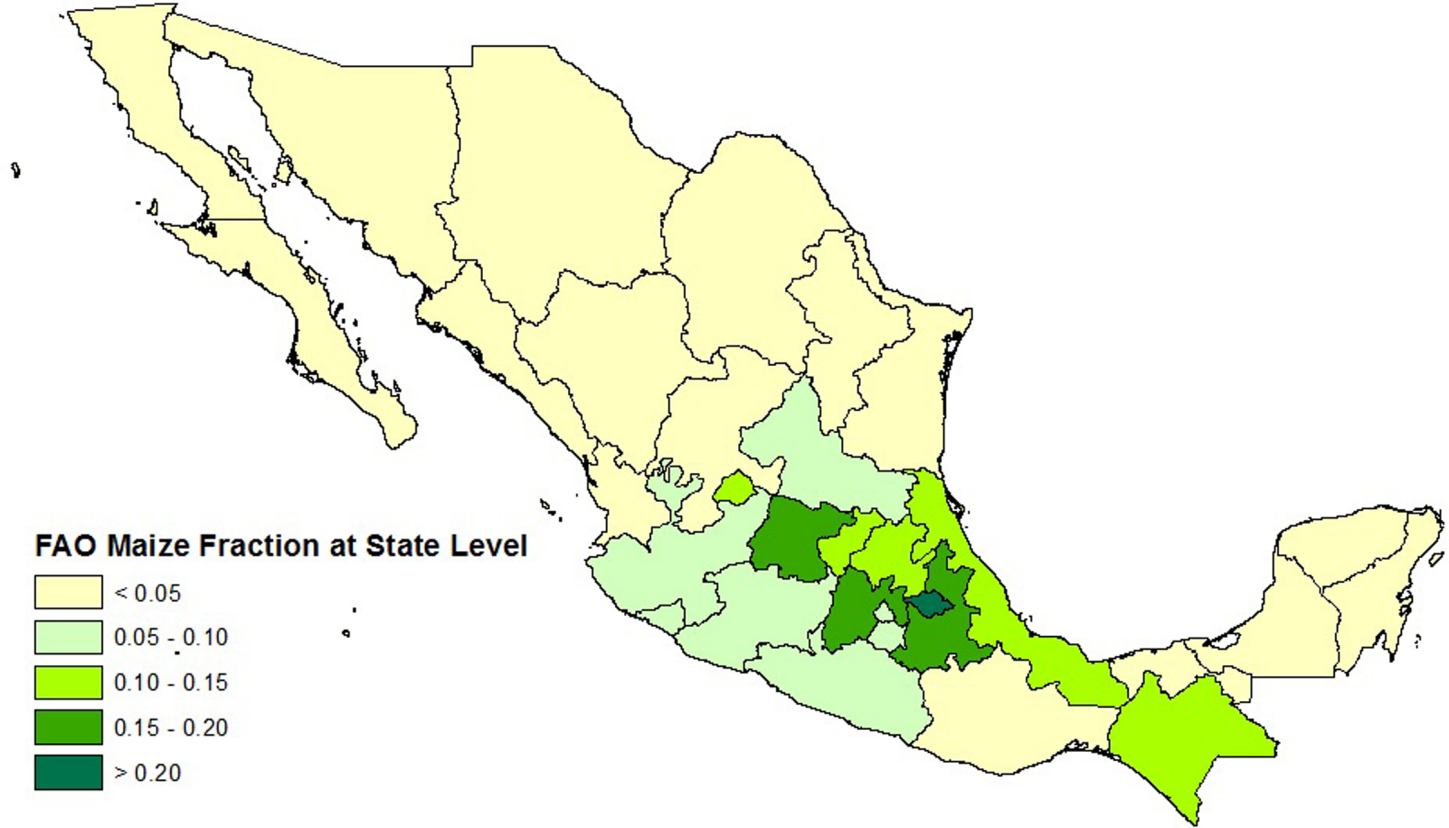
Observed FAO maize fraction data for Mexico at administrative Unit 1. The inset image illustrates the pixelated land grid.

Yet, data on cropland allocation below the national or subnational level is not widely available. Typically, cropland allocation data are collected via national census or survey instruments, and measures the total amount of land under a crop in a particular state or province. These data, however, do not usually indicate the distribution of cropland within that state. Fig 1 illustrates the issue using Mexico as an example. In the figure, the color of each state indicates the fraction of the harvested land area for maize in the state, with a darker shade of green indicating a greater harvested area in maize. The inset image shows a 5-arc minute pixelated grid placed over part of Mexico; researchers and policymakers often desire to know the distribution of the total harvested area for maize within a state over a set of pixels in the grid so that policies can be targeted where they will be most effective. Simply assuming that the pixel level allocation of cropland in a state is equal to the aggregate state level average allocation is *ad hoc*, and may mask pixel level heterogeneity that bears implications for national and international research and policy. (Various terms including gridded, parcel, pixel, and fine-scale have been used interchangeably in the literature. To avoid confusion, we use the term pixel.)

Previous economic analyses of issues related to land use have demonstrated the benefits of using cropland allocation data measured at a fine geographic scale, relative to using the state/provincial or national aggregates. For instance, Auffhammer et al. [4] describe how aggregate climate measures mask important spatial heterogeneity that is measureable using pixelated data. Hendricks et al. [5] demonstrate that county averaged data in the United States leads to qualitatively significant statistical bias in estimates of crop acreage response to price shocks, and advocate using spatially explicit pixel level data to avoid this bias. In short, pixel level data is important for research and policy related to cropland allocation because it reflects heterogeneity that is germane to land use decisions.

Yet, much of the evidence indicating advantages of pixel level data is based on analysis for the United States, where pixel level measurements are relatively abundant and are generally reliable. Researchers have lamented the dearth of pixel level measurements over a wider geographic scale; moreover, data availability and data quality problems are more severe in developing countries and regions such as Sub-Saharan Africa, where a lack of reliable data limits statistical analysis [6, 7]. In some countries, observed cropland allocation data (i.e., the data measured by national census and survey reports) is simply not available below a national or subnational (e.g., state/provincial) level. This lack of widespread availability of pixel level cropland allocation data prohibits analysis of land allocation at the pixel resolution across broad regions that researchers desire; it also limits the reliability of high spatial resolution environmental models that use land use data as inputs.

To address these issues, researchers have turned to combining census data with satellite imagery [8] or simulation methods [9] to allocate harvested areas from aggregate to pixel-level. However, these methods, discussed in detail below, are difficult to reproduce or extend in ways that allow the consumers of their outputs to assess the effects of the various empirical choices made by the authors, or update their land allocation results as updated data on harvested areas becomes available.

In this paper we suggest an alternative statistical approach that uses quasi-maximum likelihood estimation to predict the allocation of harvested area in a particular crop at a pixel level from a regression model that explains aggregated harvested areas (e.g., county or state level) in terms of pixel-level determinants of crop productivity. That is, pixel level variation in biophysical factors and the observed aggregate land allocation data drive where in each state/province the crops are located. As a first step, we use the satellite imagery data to exclude urban centers and protected areas in order to focus the allocation procedure on the cropland pixels. We then rely on the biophysical land attributes to estimate cropland use. We apply the framework to the Americas, focusing on application of the proposed approach to two cases: a single crop model for maize and a multi-crop model for maize, soybeans, and wheat.

The method we develop has the following four virtues. First, in contrast to other methods that are currently used to downscale harvested crop areas from a national or sub-national level to the pixel level [8, 9], our approach uses a novel fractional regression that is relatively simple and transparent. We believe simplicity and transparency are critical because the research that uses these downscaled predictions of cropland allocations supports policy development with deep international implications. Second, our method is relatively parsimonious, flexible, and can accommodate additional variables in the event that they become available. Third, in contrast to other methods used to downscale national and sub-national areas [8, 9], we can recover the marginal effects of the variables in the model on the allocation of cropland across the pixels within each state. Fourth, the regression framework is developed generally, and can accommodate any number of cropping options–i.e., our model allows focus on a single-crop or on multiple crops simultaneously. (Though we focus on cropland allocation, the model is also applicable to any empirical setting in which the outcome variable is a share observed at an aggregate level and the conditioning variables are observed at a disaggregated level.)

Our approach for downscaling aggregated data from censuses and other national statistics is purposefully different from the methods used by other researchers who have focused on land allocation. For instance, Ramankutty and Foley [10], Ramankutty et al. [11] and Monfreda et al. [8] combine satellite-derived land cover data with agricultural inventory data to develop a global land use database measured at the 5 arc-minute pixel level. The Monfreda et al. [8] dataset is the most comprehensive, spanning 175 distinct crops across the world, and is regarded as the standard in economic models of cropland allocation [12]. These models include large scale integrated assessment models where the focus is on disaggregated, spatially explicit measures of land use change and associated environmental and productive consequences [12–14]. Moreover, Monfreda et al.’s cropland allocations are a fundamental input into other land allocation products, such as the MIRCA dataset which characterizes global areas equipped for irrigation [15] or the global allocation of fertilizers [16].

The main drawback of Monfreda et al.’s dataset is that it is getting dated. The efforts leading to Monfreda et al.’s article were conceived as one-off initiatives that involved gathering national and subnational cropland allocation from national census and combining them with satellite imagery of land cover in order to create global maps of land allocations [17]. While the process of downscaling aggregate national or sub-national level is potentially reproducible, and the science behind it prone to improvements due to better satellite imagery and cumulative experience, we think that there is room for an alternative, statistical approach that streamlines the process of downscaling. Proposing such a statistical approach is the main objective of this paper. At the outset, we warn the reader that we do not offer a new dataset of land allocations; rather, our strategy is to use the same aggregate land allocations used by Monfreda et al., downscale them, and compare the results with the original maps of Monfreda et al. To the extent that Monfreda et al. has become a de-facto standard dataset, it seemed to us that it was the reasonable target of comparisons.

Our approach is also purposely different from You and Wood [9], who use a cross entropy approach to predict cropland allocation that relies on existing satellite-based land cover data, a broad set of biophysical and socioeconomic factors, as well as model-generated indicators of land suitability for specific crops [18, 19]. While land suitability indicators correlate with cropland allocation, and hence act as prior information of the most likely crop to be found in a given pixel, our preliminary work indicates that these indicators are largely based on biophysical attributes that are directly measurable. In contrast, our model distributes harvested areas in alternative crops observed at aggregate levels over pixels within those aggregate regions based solely on the variations in *pixel-level* biophysical attributes. In this sense, our approach is really an alternative to You and Wood [9] insofar as alternative or different sources of information can be directly incorporated as a covariate in a discrete-choice regression model. Although space prevents us from pursuing alternative experiments in this article, one can envision a case in which a researcher decides to employ a new layer of information, such as newer irrigation data or an improved set of soil attributes beyond those included here, to explore the sensitivity of the downscaled allocations. Another advantage of the regression approach to downscaling is that the researcher can explore different policy or environmental scenarios by using the parameter estimates of the model to predict land allocations under different conditions, e.g., under a warmer climate with and without irrigation. (Such endeavors are part of our current research program.)

Our approach is closest to Li et al. [20], who estimated land allocation as a function of geo-referenced biophysical factors–some of which include crop-specific land suitability variables–and spatially explicit producer prices for the Democratic Republic of Congo. Relative to our model, their approach has two main limitations. First, they use a discrete outcome logit regression model that restricts the allocation within each pixel to a single land use. Second, their model adopts pixel (1 km by 1 km) measurements that are not widely available, rendering application of their model to a larger geographic area challenging.

The rest of the paper proceeds as follows. Section Theoretical framework describes our approach. We formalize the econometric problem and develop a robust quasi-maximum likelihood framework based around a modified fractional logit regression. In Section Data and empirical models, we illustrate our approach by developing empirical models of cropland allocation for maize as a single crop example and for multiple crops (maize, soybeans, and wheat) simultaneously as a multiple crop example across North, Central and South America. Section Data and empirical models also describes the data. Section Parameter estimates and marginal effects presents our statistical results including the marginal effects of pixelated data on predicted cropland allocation. Section Model validation conducts both in-sample and out-of-sample validation exercises to assess the predictive performance of the model. We also provide evidence that our approach is capable of reliably predicting cropland allocation at the pixel level. In Section Illustrating the pixel-level predictions we demonstrate how the model can be used to develop the pixel level cropland allocation data, and Section Conclusions provides conclusions.

## Theoretical framework

### Model preliminaries

We define a land parcel measured at the 5 arc-minute longitude/latitude level as a pixel. Basic biophysical land attributes such as temperature, precipitation, and soil pH are available at the pixel level; one important reason why these data are more readily available is that they are collected from globally positioned monitoring stations. Agricultural land use is observed at a more aggregated level–typically, the total area of cropped land and the share of cropped land devoted to a particular crop, observed at the subnational administrative unit level (e.g., the state or provincial level). Formally, we will call the state or provincial level Administrative Unit Level 1, and we will call the district or county level Administrative Unit Level 2. These data come directly from national census or survey instruments. These subnational administrative units are composed of pixels (as in Fig 1). Our goal is to estimate the fraction of each individual pixel that is cropped in a particular crop given the available pixel level biophysical measurements and aggregate land shares.

Papke and Wooldridge [21] propose a quasi-maximum likelihood estimator to analyze models with fractional response; Mullahy [22] extends the model to the case of multivariate fractional response. Maximizing a Bernoulli log-likelihood function produces consistent estimates of the structural parameters, and a logistic function can ensure that the fitted values are restricted to the unit interval [23, 24]. Our case is similar to Papke and Wooldridge [21] and Mullahy [22], but not identical. The difference is that our conditioning variables are measured at the pixel level while the outcome is observed at the administrative level, which is more aggregated than the pixel level. In this structure, the outcome does not vary at the same level as the regressors, and aggregation is required.

To address this issue, we develop an aggregated fractional response model to accommodate the structure of the problem. The added value is that the model is capable of predicting cropland allocation at any level of spatial resolution at which the conditioning variables are measured or aggregated, regardless of whether the outcome is observed at that level.

### Econometric model and estimation

Formally, let *j* index administrative units for j={1,2,…,J} and *k* represent crops for k={1,2,…,K}. (We derive the model in terms of crops, but note that this econometric model generally fits any context with a similar data structure. In our empirical models, the *k = 1* denotes any land use other than the crops of interest (excluding protected area and urban area), in other words, it represents all crops not included in K={2,…,K}.) Let $y_{jk}$ be the observed fraction of land area in administrative unit *j* that is in crop *k*, such that $0\leq y_{jk}\leq 1$; note also that for each *j*, $\sum_{k=1}^{K}y_{jk}=1$. Let $Z_{ijk}$ be the (unobserved) fraction of cropped land in pixel *i* in administrative unit *j* that is cropped in crop *k*, where $i=\{1,2,\ldots ,I_{j}\}$ is the pixel index that allows the total number of pixels in each administrative unit to vary. Define $X_{ij}$ to be an *N*-dimensional vector of observable biophysical attributes for pixel *i* in administrative unit *j*.

We are interested in estimating the parameters *β* in the conditional mean for pixel level share $Z_{ijk}$:

$$E\left[Z_{ijk}\text{|}X_{ij}\right]=G_{ijk}\left(W_{ij}(X_{ij}\right),\beta_{k})$$ (1)

where $W\left(∙\right):R^{N}\to R^{M}$ reflects transformations of the fundamental explanatory variables (linear, quadratic, interaction, etc.), $G\left(\cdot \right):R^{M}\to R,0\leq G\left(\cdot \right)\leq 1$ is a function that maintains the unit interval restriction on the conditional mean. We parameterize G(⋅) using a logistic function, and the predicted fraction of land in crop *k* in pixel i in administrative unit *j* becomes:

$$G_{ijk}\left(W_{ij}(X_{ij}),\beta_{k}\right)=\frac{\text{exp}\left(W_{ij}(X_{ij}\right)\beta_{k})}{\sum_{i=1}^{K}\text{exp}\left(W_{ij}(X_{ij}\right)\beta_{k})}\;\text{where}\;\beta_{1}=0.$$ (2)

The *β*_*1*_ = 0 normalization facilitates parameter identification relative to a base cropland allocation. We extend Eq (2), which is defined at the pixel level, to the administrative unit level via aggregation–i.e., the predicted fraction of land in crop *k* in administrative unit *j* is equal to the average pixel fraction weighted by area. The predicted fraction of land in crop *k* in administrative unit *j* is

$$H_{jk}=\frac{\sum_{i\in I_{j}}G_{ijk}\left(W_{ij}(X_{ij}),\beta_{k}\right)A_{ij}}{\sum_{i\in I_{j}}A_{ij}}$$ (3)

where $A_{ij}$ is the area of pixel *i* in state *j*. Function (3) aggregates our predicted land shares for pixel *i* to the administrative level, hence converting pixel level information to the administrative level so that the pixel level land attribute (biophysical) data can be used to explain cropland allocation. Given $H_{jk}$ the quasi-log-likelihood function to be maximized with respect to the parameters *β*_*k*_ is:

$$L=\sum_{j=1}^{J}\sum_{k=1}^{K}y_{jk}\;\ln\;H_{jk}.$$ (4)

In addition to being relatively simple to optimize to obtain parameter estimates, the quasi-maximum likelihood estimator is consistent regardless of the conditional distribution of *y* given *x* as long as the conditional mean is correctly specified and the distribution is a member of the linear exponential family [21, 23]. This makes the estimator relatively robust. This method is generally applicable to other cases in which only aggregate level data is available for the outcome, but pixel level estimates are desired. This framework may also be adapted to a panel data context using a correlated random effects approach [25].

Gourieroux et al. (1984)[23] and Wooldridge [24, 26] derive the asymptotic variance of the quasi-maximum likelihood estimator using the estimated information matrix and the outer product of the score. Papke and Wooldridge [21] and Mullahy [22] provide expressions for the univariate and multivariate fractional logit models; we follow the same asymptotic variance calculation but base the formulation at the aggregated state level $H_{jk}$. The estimated asymptotic variance of $\beta_{k}$ is the diagonal of:

$$F^{-1}BF^{-1}/J$$ (5)

where $F^{-1}$ denotes the inverse Hessian and *B* denotes the outer product of the score. (An alternative is to use a block bootstrap that preserves the pixel to state ratio across the bootstrap replications (i.e., sample all pixels from each bootstrap sampled states). The authors’ calculations have shown this bootstrap procedure yields standard errors that are virtually identical to those from the asymptotic variance-covariance formulation.)

### Software implementation

It is straightforward to implement our estimators. The most straightforward means of implementation is to use our open access tool that deploys this estimator, and is available at https://mygeohub.org/tools/flat. Source code and data used in this paper are free to download via the tool user interface. Alternatively, estimation can be conducted via any numerical optimizer. In our own research, we use both the General Algebraic Modeling System (GAMS; version 24.1.1) [27] and R (version 3.1.0) [28] to solve the log-likelihood in Eq (4) for the parameter values and covariance matrix. Specifically, we use the CONOPT solver in GAMS that solves the log-likelihood via numerical computation of the gradients using a quasi-Newton optimizer, and in R we use the “optimx” package to call the BFGS optimizer to solve the log-likelihood function this time using analytical gradients. Both setups yield virtually identical parameter estimates, with the relative error on the order of 1E-8.

### Cropland allocation, marginal effects, and policy analysis

An important difference in our approach to predicting cropland allocation is that we use variation in pixel level biophysical measurements to generate our predictions, rather than relying on satellite image derived crop shares for each pixel (e.g., Monfreda et al., [8]). This gives us two unique advantages. First, we project cropland allocation based on pixel level biophysical factors and aggregate cropland statistics, which allows for estimation when satellite imagery is not available or is unable to distinguish between crop types.

Second, the econometric approach allows us to derive the effects of marginal changes in pixel level biophysical factors on cropland allocation. The marginal effect on the fraction of cropland in crop *k* in pixel *i* in state *j* with respect to regressor $X_{ijm}$ is

$$\frac{\partial G_{ijk}\left(W_{ij}(X_{ij}\right),\beta_{k})}{\partial X_{ijm}}.$$ (6)

The exact form of this marginal effect depends on the transformations in ${W_{ij}(X}_{ij})$ and the specification of the index function.

Given the logistic parameterization of the likelihood model, we also calculate the odds ratio. Holding everything else constant, an odds ratio greater than one means that a one unit change in ***X*** would make the fraction of land in crop *k* larger, while an odds ratio less than one means that a one unit change in ***X*** leads to a decrease in the fraction of land in the crop. For the simple case that a variable enters linearly into the index function, the odds ratio is $exp(\beta_{km})$. The calculation details for marginal effects and odds ratios including non-linear effects due to second-order and interaction terms can be found in Song [29].

#### Remark 1

We have described the pixel level measurements as biophysical factors, though clearly, economic and institutional forces also have a substantial impact on land allocation and crop choice. Our model structure does not preclude the inclusion of additional variables. Currently, we are operating under the constraint that straightforward measurements of these other factors are not available at a pixel level across a broad geographic area, either because the data do not exist, or because factors do not vary at a pixel level (such as national or state laws, institutional structures, and in many cases, prices). This constraint is not unique to our work. We control for these additional factors to the best of our ability using national level indicators (see Section Data and empirical models).

#### Remark 2

The parameter estimates and pixel level predictions are obtained under the assumption that factors related to political and economic differences (e.g., laws, agricultural policies, consumer preferences, etc.) are held constant via the indicators. Likewise, the marginal effects should be interpreted as a (potential) re-allocation of cropland induced by changes in biophysical measurements, holding the country level factors constant.

## Data and empirical models

We develop two empirical applications: a single-crop model for maize, representing the case in which one is interested only in whether land is dedicated to a crop or some other use, and a multi-crop model of maize, soybeans, and wheat, for the case in which one is interested in multiple crops. For both models, we focus on harvested crop area spanning North, Central and South America. The 5 arc-minute resolution is a commonly used pixel measurement [30, 31], and yields pixels of about 100 square kilometers at the equator, 60 square kilometers in Minnesota, and nearly zero square kilometers near the north/south pole. S1 Table in the supplementary section provides a comprehensive list of all data we employ, including the units of measurement and source.

### Harvested land area

We use data series of built-up land and protected areas to exclude pixels that are in urban centers or protected areas such as national parks and forests. Total area in a pixel, $A_{ij}$, is calculated based on longitude and latitude. The total land area in an administrative unit is collected from Statoids (see S1 Table in the supplementary section). We restrict the sample to administrative units with at least 0.5 percent total land area in maize for the maize model and at least 0.5 percent total land area in each of the three crops for the multi-crop model. This excludes administrative units where area in these crops is negligible. We also require that the sum over the shares of cropland in maize, soybeans, and wheat does not exceed 1 for the multi-crop model. (There are a few states for which the sum of land shares in maize, soybeans, and wheat is greater than 1. This can be a result of multiple cropping seasons, or data inconsistencies. We leave these issues for future research.)

The total areas of land harvested in maize, soybeans, and wheat at Administrative Unit Level 1 are collected from FAO Agro-MAPS (see S1 Table in the supplementary section), which is the largest source of subnational agricultural harvested land area data [8]. These data come from national or subnational census or survey statistics. In these data, the years for which harvested area data is available vary across countries; to build our sample we choose the years that are closest to the year 2000 to form a circa 2000 dataset (Ramankutty et al., [11] also use a circa 2000 dataset). For example, state level harvested maize area data for the United States is not available for the year 2000 from Agro-MAPS, so instead we use 2001 data. For most of the countries in our analysis the data measurements come either from the year 2000/2001 or from the mid-late 1990s; the largest difference is for Costa Rica, for which the data is measured in 1984. The gaps in these data arise because the national censuses and surveys are typically done at five year intervals. Further, not every administrative unit has data for maize, soybeans, and wheat, and not every unit is reported in Agro-MAPS. Hence we only include units with reported FAO data. Since there are a few cases where the names of the units have changed in recent years or a unit has been divided into separate units, we verify the administrative divisions using the CIA World Factbook and Global Administrative Areas (GADM) database (see S1 Table in the supplementary section) to ensure a consistent set of administrative units for circa 2000.

### Pixel level biophysical data

Our model includes key biophysical variables that influence crop choice. Climate is captured by long-term average (1961–1990) temperature (in degree Celsius) and precipitation (meters/year, original data was in millimeters/year) over the growing season. These data from part of the IIASA/FAO [32]’s assessment of Global Agroecological Zones, and were constructed using the time-series climate anomalies from New et al. [33]. We also include elevation (in thousands of meters above sea level, original data was in meters), obtained from TerrainBase, a global model of terrain and bathymetry on a regular 5-minute grid [34]. Indicators of soil quality are captured by soil pH level (0–14) and soil carbon content (kg per square meter to 1 meter depth) both from the SoilData System developed by the Global Soils Data Task from the International Geosphere-Biosphere Program [35]. In addition, we include land slope (from almost flat to steep, 0.0025–0.725), and latitude. Elevation and latitude data are at the 5-minute degree level; other variables are available at a 30 arc-minute resolution and are downscaled assuming all the 5-minute degree cells within a 30-minute degree cell have the same value. Our preferred specification is quadratic in temperature, precipitation, and latitude.

We also include the interaction between temperature and precipitation; this specification of temperature and precipitation is similar to that used by Lobell et al. [36], Lobell et al. [37], Schlenker et al. [38], and Schlenker and Roberts [39]. We define soil pH to be the soil pH deviation from pH6.5 as pH6.5 is approximately optimal for maize, soybeans, and wheat [40, 41] (http://www.cropnutrition.com/efu-soil-ph#soil-acidity. Accessed on Aug 30, 2015.). We expect the fraction of a pixel that is cropped in maize to decrease with an increase in the deviation (above or below) of soil pH from the optimal pH6.5 level. To allow the response above pH6.5 and below pH6.5 to be asymmetric, we include two variables max(pH6.5-pH, 0) and max(pH-pH6.5, 0). Table 1 reports descriptive statistics for the variables, divided into the North, Central and South American regions. All the statistics for the variables described above can be downloaded for free as part of the Atlas of the Biosphere data series, which can be accessed via the Center for Sustainability and the Global Environment (SAGE), Nelson Institute for Environmental Studies, University of Wisconsin-Madison website (http://nelson.wisc.edu/sage/data-and-models/atlas/about.php) and the IIASA/FAO GAEZ website (http://gaez.fao.org/Main.html#). We also list the detailed data source information for each variable in S1 Table in the supplementary section.

**Table 1:** Descriptive statistics of the biophysical variables measured at the pixel level.

	Variable	Mean	Standard Deviation	Min	Max
*North America*	Temperature	16.660	6.087	1.833	28.667
	Precipitation	0.813	0.361	0.068	3.258
	Elevation	0.569	0.601	-0.227	3.704
	Max(pH6.5-pH,0)	0.466	0.617	0.000	2.300
	Max(pH-pH6.5,0)	0.342	0.466	0.000	1.665
	Soil Carbon	6.351	2.895	1.608	22.356
	Slope	0.056	0.065	0.003	0.375
	Latitude	37.326	9.612	14.625	56.792
*Central America*	Temperature	23.474	2.514	15.833	27.500
	Precipitation	2.256	0.739	1.142	4.678
	Elevation	0.674	0.609	-0.197	3.300
	Max(pH6.5-pH,0)	0.611	0.422	0.000	1.400
	Max(pH-pH6.5,0)	0.030	0.101	0.000	0.640
	Soil Carbon	6.900	1.740	3.984	12.724
	Slope	0.134	0.088	0.003	0.375
	Latitude	13.263	2.156	7.292	16.042
*South America*	Temperature	22.493	3.967	-0.167	28.500
	Precipitation	1.181	0.494	0.000	5.667
	Elevation	0.564	0.709	-0.132	4.783
	Max(pH6.5-pH,0)	0.690	0.609	0.000	1.869
	Max(pH-pH6.5,0)	0.194	0.392	0.000	1.563
	Soil Carbon	5.147	1.646	1.325	13.183
	Slope	0.054	0.065	0.003	0.375
	Latitude	-18.537	11.908	-40.958	11.458

We follow the CIA World Factbook (https://www.cia.gov/library/publications/the-world-factbook/) division of the Americas. Our data includes the North American countries Canada, United States and Mexico; the Central American countries Costa Rica, Guatemala, Honduras, Nicaragua and Panama; the South American countries Argentina, Bolivia, Brazil, Chile, Colombia, Ecuador, Paraguay, Peru, Uruguay and Venezuela.

### Indicator variables and final sample

We add binary indicators for each country included in the analysis (see Table 2 for included countries), with a group of South American countries (Ecuador, Peru, Paraguay, and Uruguay) excluded as the base group for the maize model. These indicators allow us to account for political and economic factors that influence crop production but are unobservable and/or do not vary within each country. For the multi-crop model, we include country indicators for Argentina and the United States and use the rest of the countries as the base. (Five countries are included in the multi-crop model based on the sample selection criteria. Different model specifications show that the model with Argentina and United States indicators yields the lowest root mean squared error.)

**Table 2:** List of countries in each model with the number of administrative units in the sample.

*Maize Model*			
Argentina (9)	Bolivia (3)	Brazil (18)	Canada (1)
Chile (3)	Colombia (6)	Costa Rica (2)	Ecuador (10)
Guatemala (12)	Honduras (14)	Mexico (29)	Nicaragua (17)
Panama (5)	Peru (8)	Paraguay (13)	Uruguay (5)
United States (28)	Venezuela (13)		
*Multi-crop Model*			
Argentina (8)	Brazil (3)	Mexico (2)	Paraguay (5)
United States (22)			

In the maize model, we include only the states in which there is at least 0.5 percent cropland in maize. In the multi-crop model, we require each state to have at least 0.5 percent cropland in each crop. The numbers indicated in parentheses after the country names are the number of states included for the country.

Based on our selection criterion, 196 administrative units from 18 countries are included in the maize model. These countries are listed in Table 2 with the number of states included from each country in parentheses. For the multi-crop model, a total of 40 states from 5 countries are included.

## Parameter estimates and marginal effects

### The single-crop model

**Fig 2:**
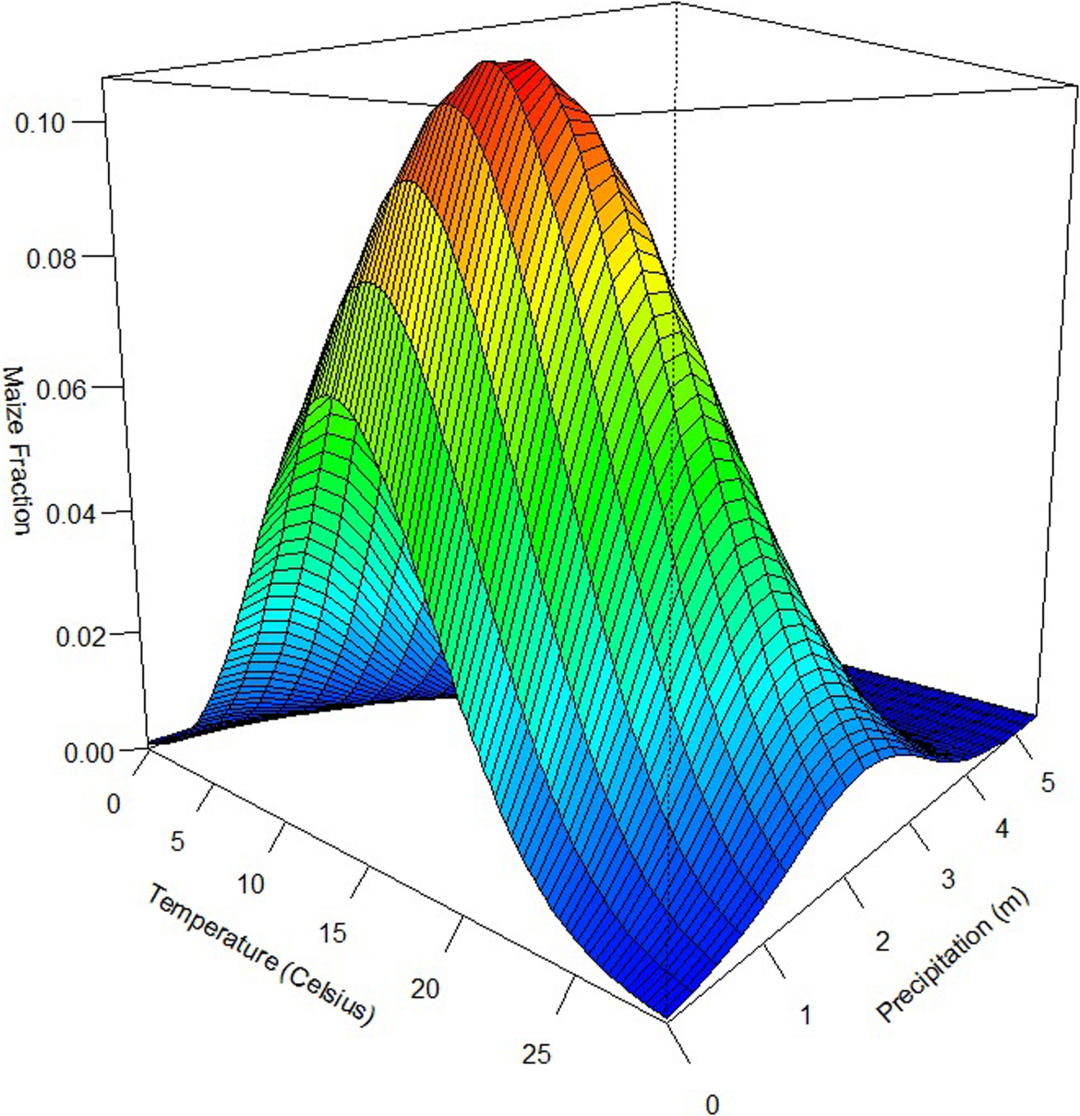
Estimated relationship between temperature, precipitation, and the fraction of maize for the single-crop maize model (holding the other variables constant at their means).

The coefficient estimates and standard errors for the maize model are reported in Table 3, and the implied marginal effects and odds ratios are reported in Table 4. Following Greene [42], we assess statistical significance in our model via the t-values on the parameters, and then report and draw conclusions directly from the implied marginal effects. These coefficient estimates and marginal effects can be immediately deployed in a variety of policy analysis contexts to understand how changes in biophysical factors might influence cropland allocation. Temperature, precipitation, soil pH (both deviations below and above pH6.5), soil carbon, slope, and latitude are all statistically significant. (Hypothesis tests indicate that the coefficients on the deviations above and below pH6.5 are statistically different at the 0.05 level, which confirms our choice to specify above/below pH6.5 separately.) Temperature has a positive impact on the fraction of maize in a pixel. The average temperature of the growing season in our sample is 19.34 degrees Celsius. All else equal, a one degree Celsius increase in temperature above this average increases the fraction of maize by about 0.35 percent (Table 4). The average precipitation in our sample is 1 meter. All else equal, a one meter increase in precipitation increases the maize fraction by about 1.03 percent. In Fig 2 we plot the relationship between temperature, precipitation, and the predicted fraction of maize, evaluating all observations at the base group for the country indicators while holding other variables constant at their mean. The average growing season temperature ranges from -0.17 to 28.67 degrees Celsius and the average precipitation is between 0 and 5.67 meters. At low levels of precipitation, as temperature increases the maize fraction first increases and peaks around 10 degrees Celsius, then decreases to about 0. Holding temperature constant, the maize fraction increases first, then drops to about 0. At low levels of precipitation, an increase in precipitation leads to a rapid increase in the maize fraction, while at higher levels of precipitation, a further increase in precipitation does not affect the maize fraction.

**Table 3:** Quasi-maximum likelihood estimates and standard errors for the maize model.

Variable	Coefficient	Standard Error
Intercept	-7.155***	1.949
Temperature	0.582***	0.158
Temperature Squared	-0.021***	0.004
Precipitation	-0.578	0.713
Precipitation Squared	-0.584***	0.141
Temperature∙Precipitation	0.114***	0.028
Elevation	-0.062	0.281
Max(pH6.5-pH,0)	-0.923***	0.203
Max(pH-pH6.5,0)	-1.622***	0.383
Soil Carbon	0.157***	0.047
Slope	-6.142**	2.289
Latitude	0.044**	0.015
Latitude Squared	0.0004	0.001
Argentina Indicator	0.392	0.300
Bolivia Indicator	0.611	0.516
Brazil Indicator	1.432***	0.300
Canada Indicator	-3.965***	1.277
Chile Indicator	0.377	0.527
Colombia Indicator	-0.333	0.327
Costa Rica Indicator	-0.743*	0.439
Guatemala Indicator	1.155*	0.578
Honduras Indicator	0.131	0.539
Mexico Indicator	0.450	0.641
Nicaragua Indicator	0.741	0.462
Panama Indicator	0.052	0.462
United States Indicator	-1.887	1.150
Venezuela Indicator	0.500	0.480

*p < 0.05;

**p < 0.01;

***p < 0.001

**Table 4:** Implied marginal effects and odds ratios for the single-crop maize model.

Variable	Marginal Effect	Odds Ratio
Temperature	0.004	0.890
Precipitation	0.010	1.120
Elevation	-0.005	0.940
Max(pH6.5-pH, 0)	-0.079	0.397
Max(pH-pH6.5, 0)	-0.138	0.198
Soil Carbon	0.013	1.170
Slope	-0.524	0.002
Latitude	0.106	2.567

The marginal effects and odds ratios are averages over all included pixels.

Deviation from the optimal soil pH of 6.5 decreases the fraction of maize. A decrease in pH below pH6.5 reduces the maize fraction by about 7.87 percent, and an increase in pH above pH6.5 reduces the fraction of maize by about 13.83 percent. Average soil carbon content is 5.83 kg per square meter; the maize fraction increases by about 1.34 percent as soil carbon content increases by 1 kg per square meter. Slope has a negative impact on the fraction of maize, such that as land becomes steeper, the maize fraction decreases. Finally, there is a positive correlation between latitude and the fraction of maize–the fraction of land in maize is larger at a higher latitude.

**Fig 3:**
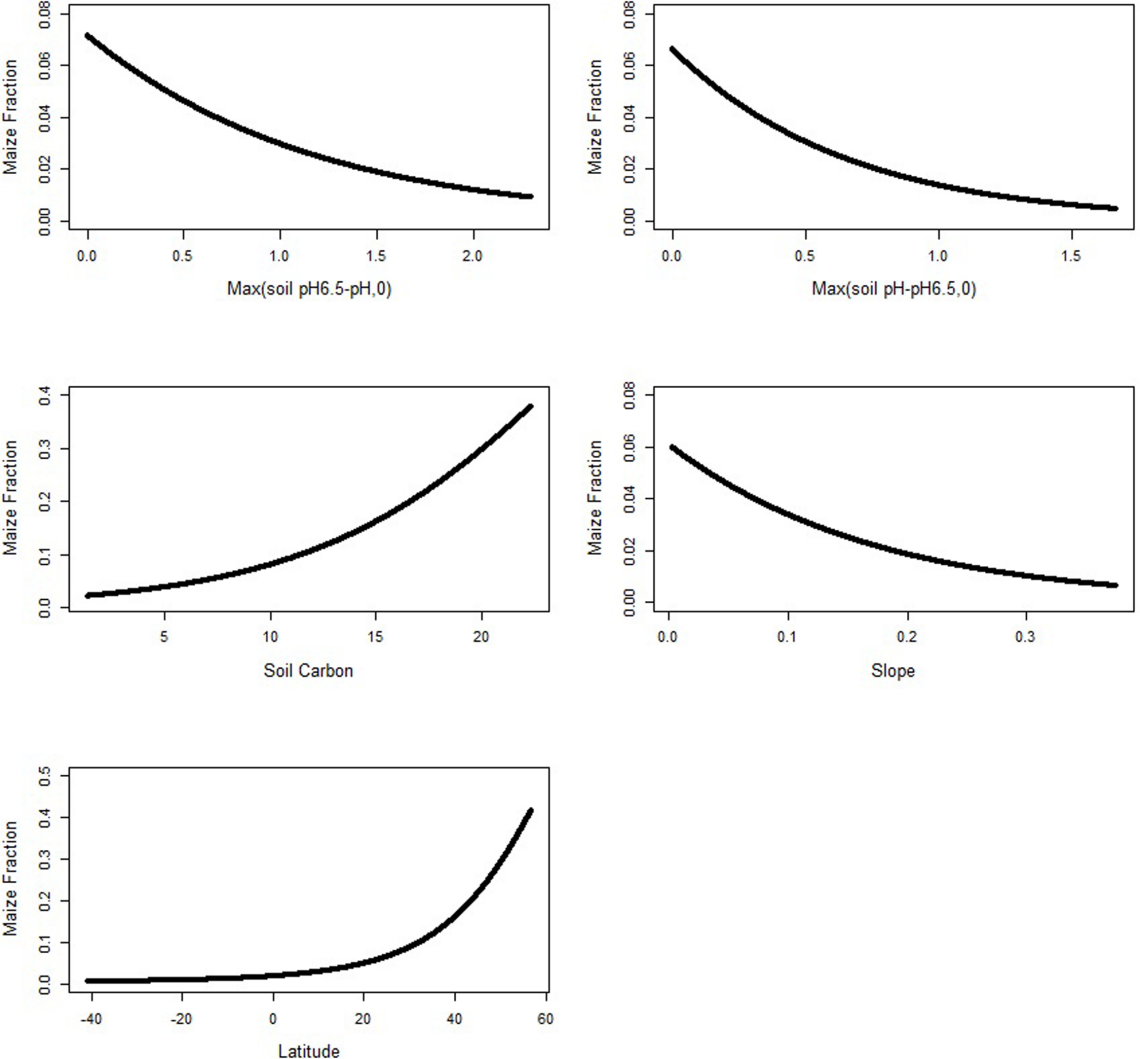
Estimated relationship between elevation, soil pH, soil carbon, slope, latitude and the fraction of maize for the single-crop maize model (holding the other variables constant at their means).

To further investigate the relationships among the variables and the fraction of maize, we plot the maize fraction as a function of each variable, holding the other variables constant at their means (see Fig 3). Pixel level soil pH ranges from 4.20 to 8.17, and deviation from above and below the optimal soil pH of 6.5 leads to a decrease in the maize fraction. Latitude ranges between -40.96 and 56.79 degrees. We see an increase moving from South to North America.

### The multi-crop model

Similar to the maize model, we report the coefficient estimates for each regressor in the multi-crop model in Table 5. The significance of these variables varies across crops. Temperature and precipitation are significant for maize and soybeans. Temperature squared and the interaction between temperature and precipitation are significant for all three crops. Precipitation squared is significant for soybeans, but not for maize and wheat. The land attribute variables–slope and soil pH above pH6.5 –have a significant impact on the crop fraction for all three crops. Elevation, soil pH below pH6.5, and latitude, have a significant impact on the maize and soybean fractions. Implied marginal effects and odds ratios are displayed in Table 6.

**Table 5:** Quasi-maximum likelihood estimates and standard errors for the multi-crop model.

Variable	Maize	Soybeans	Wheat
Intercept	9.663 (11.080)	18.725* (8.968)	5.883 (14.440)
Temperature	2.877*** (0.846)	2.715*** (0.647)	1.089 (0.752)
Temperature Squared	-0.118*** (0.032)	-0.114*** (0.026)	-0.058* (0.031)
Precipitation	-20.750** (8.699)	-26.153*** (7.103)	-15.314 (9.574)
Precipitation Squared	2.825 (2.097)	4.494** (1.636)	0.506 (2.008)
Temperature∙Precipitation	0.687* (0.401)	0.707 (0.352)	0.672* (0.395)
Elevation	-4.499** (1.639)	-7.289*** (1.520)	-1.331 (2.743)
Max(pH6.5-pH,0)	-1.960*** (0.320)	-2.236 *** (0.260)	-0.669* (0.348)
Max(pH-pH6.5,0)	-3.452** (1.130)	-2.816** (1.144)	-0.247 (0.778)
Soil Carbon	0.144 (0.143)	0.076 (0.155)	0.125 (0.132)
Slope	-16.314* (8.647)	-23.694** (9.853)	-26.537* (14.671)
Latitude	0.317*** (0.060)	0.340*** (0.041)	0.072 (0.060)
Latitude Squared	-0.007** (0.002)	-0.008*** (0.002)	-0.003 (0.004)
Mexico Indicator	-1.286*** (3.199)	-1.641*** (2.406)	-0.710 (3.359)
United States Indicator	-19.862*** (4.011)	-21.262*** (2.740)	-4.680 (3.710)

*p < 0.05;

**p < 0.01;

***p < 0.001

**Table 6:** Implied marginal effects and odds ratios from the multi-crop model for maize, soybeans, and wheat.

Variable	Maize		Soybeans		Wheat	
	Marginal Effect	Odds Ratio	Marginal Effect	Odds Ratio	Marginal Effect	Odds Ratio
Temperature	-0.012	0.733	-0.025	0.759	-0.005	1.183
Precipitation	0.204	3.902	-0.226	32.838	-0.013	2.779
Elevation	0.222	10.066	-0.440	0.038	-0.002	70.630
Max(pH6.5-pH, 0)	0.007	0.891	-0.079	0.519	-0.005	3.065
Max(pH-pH6.5, 0)	-0.097	0.347	-0.006	1.199	0.005	9.547
Soil Carbon	0.008	1.095	-0.005	0.982	0.002	1.065
Slope	0.561	18.782	-1.220	0.0002	-0.375	0.0001
Latitude	0.016	1.370	0.011	1.375	0.002	1.128

The marginal effects and odds ratios are averages over all included pixels.

Temperature has a negative impact on the fraction of all three crops on average within the studied area. The average temperature of the growing season in our sample of administrative regions is 18.39 degrees Celsius. A one degree Celsius increase in temperature above this average decreases the fraction of maize by 1.19 percent, soybeans by 2.48 percent, and wheat by 0.51 percent, indicating that changes in temperature have a larger impact on maize and soybean fractions than on wheat. Precipitation has a negative impact on the fractions of soybeans and wheat, but a positive impact on maize. Specifically, a one meter increase in precipitation from the average level of 0.95 meters leads to an increase in the fraction of maize of 20.41 percent, and a decrease in the fraction of soybeans of 22.57 percent, and wheat of 1.34 percent. Deviation in soil pH from pH6.5 has a negative impact on maize, soybeans, and wheat, and slope has a negative relationship with soybeans and wheat. Latitude, on average, has a positive relationship with the fractions of all three crops, meaning that as we move north to states in the Northern United States, crop fractions tend to increase. The calculated odds ratios imply similar results as the marginal effects.

**Fig 4:**
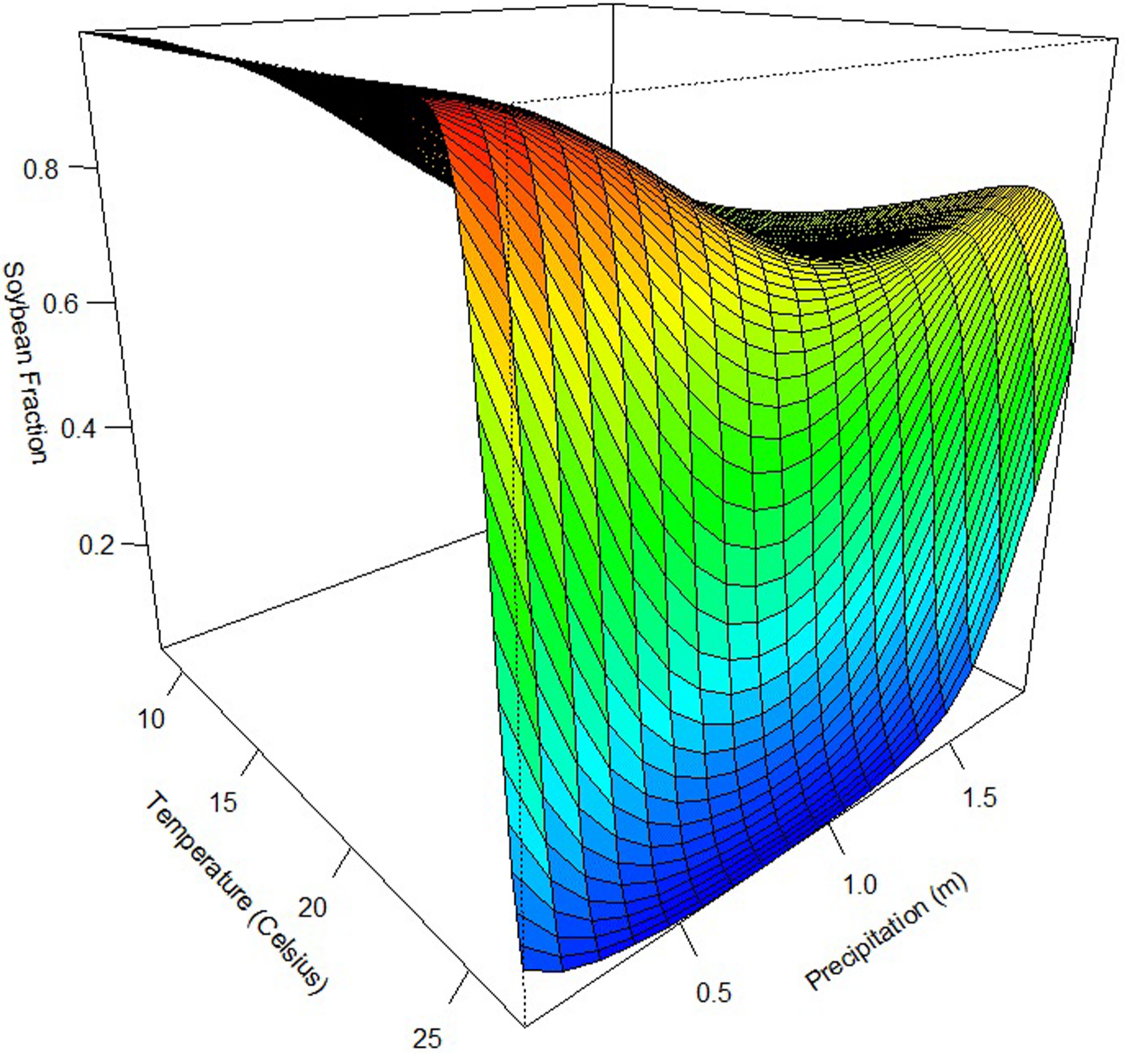
Estimated relationship between temperature, precipitation, and the soybean fraction for the multi-crop model (holding the other variables constant at their means).

Fig 4 shows a 3-dimensional plot of the relationship between temperature, precipitation, and the soybean fraction. The lowest average temperature in the growing season across all included states is 8.33 degrees Celsius, and the highest is 27.00 degrees Celsius. As temperature increases, the soybean fraction first increases and decreases slightly, then increases and decreases; the minimum level of average annual precipitation is 0.07 meters, and the maximum is 1.94 meters; as precipitation increases, the soybean fraction decreases then increases.

**Fig 5:**
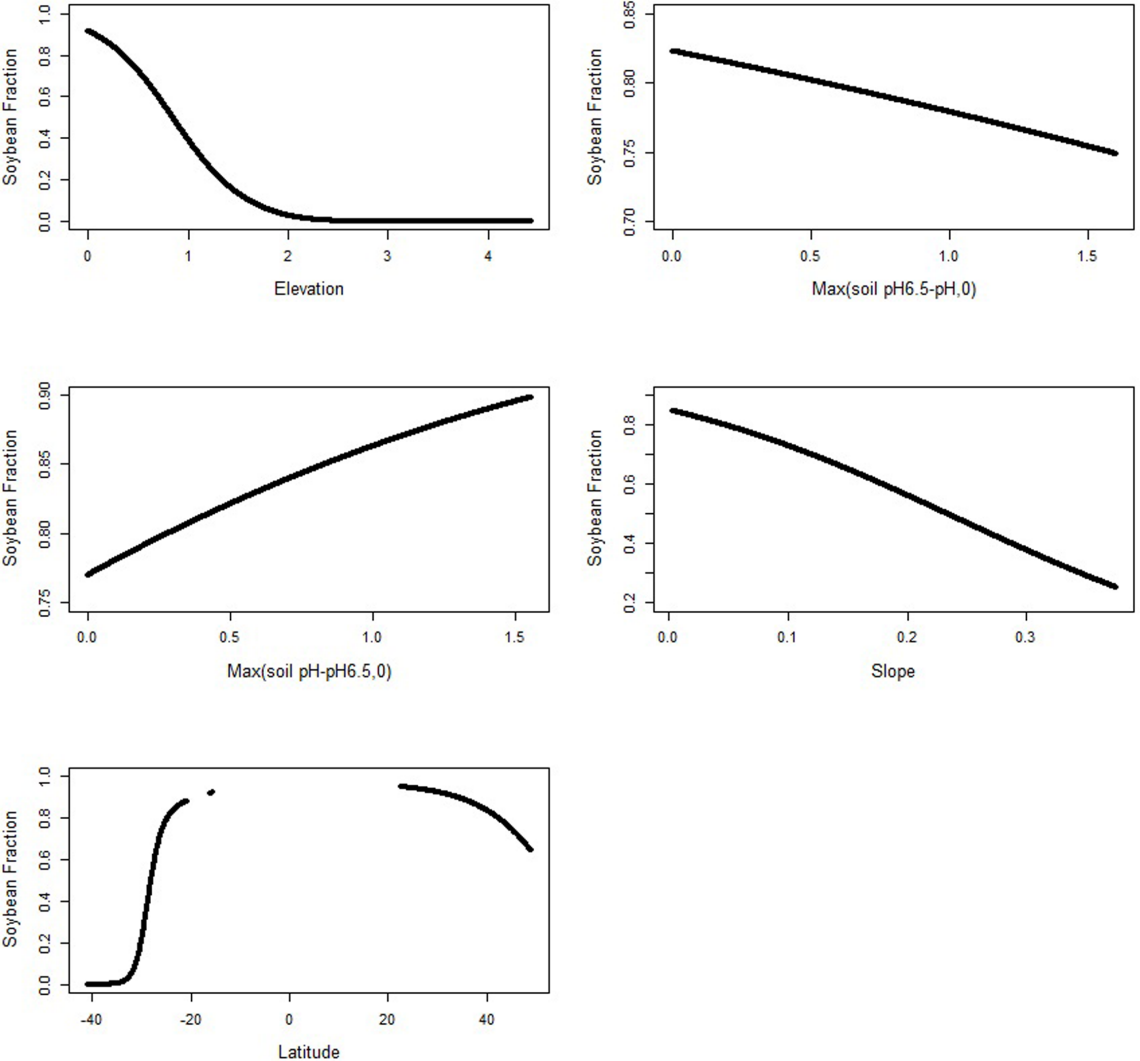
Estimated relationship between elevation, soil pH, slope, latitude, and the soybean fraction for the multi-crop model (holding the other variables constant at their means).

Graphical illustrations for the other variables that have statistically significant impacts on the soybean fraction are shown in Fig 5. Elevation has a negative impact on soybean fraction. The minimum soil pH is 4.90. Deviation from below the optimal soil pH of 6.5 leads to a decrease in the soybean fraction. The maximum soil pH is 8.05. Soybean fraction increases in the studied area when soil pH deviates from 6.5. Slope has a negative impact on the soybean fraction: as land gets steeper the soybean fraction decreases. Latitude has a positive overall impact on the soybean fraction. Moving towards the Northern Hemisphere, the soybean fraction first increases, then stays high around the Central American countries and Mexico, then decreases moving further north passing the United States Corn Belt. For visualization purpose, we plot smooth lines/surfaces instead of the direct estimates in Figs 2–5, taking continuous values in between the maximum and minimum values of each variable of interest. For the multi-crop model, we do not have data between latitude -15 and +20, therefore, we leave a gap in the latitude-fraction plot to reflect this.

## Model validation

### In-sample and out-of-sample validation

In general, the estimated relationship between the biophysical variables and the crop fraction within each pixel is consistent with our expectation. We now validate the predictive power of our model, considering both in-sample and out-of-sample validation. By out-of-sample validation, we mean that we use our parameter estimates that rely on Level 1 data to predict the crop fractions at the more disaggregated Level 2. Such predictions are then contrasted with observed cropland shares at the level 2. Ideally, Level 2 data should add up to Level 1 values. However, we do not have complete Level 2 data for all counties/districts, but using the available Level 2 data enables us to validate prediction at a spatial level that is finer than the level of data that we use to estimate the model. To validate, we compare the crop fraction predicted by our model to the actual crop fraction reported by FAO Agro-MAPS at both Administrative Levels 1 and 2. Level 2 FAO data are available for only 108 states for the maize model, and 32, 35, and 35 states for the multi-crop model for maize, soybean, and wheat. USDA NASS Quick Stats includes more Level 2 units (counties) in their database for the United States; for Level 2 validation for the United States, we rely on USDA NASS data instead of Agro-MAPS Level 2 data.

The predicted fraction of a crop in each pixel comes from Eq (2) given the estimated parameters. The predicted harvested area for a crop in a pixel equals the total land area in the pixel times the fraction of the pixel that is in that crop. Summing over the predicted crop area in all the pixels in each Level 1 or 2 unit yields the total predicted area in that crop for the unit. To create the validation plot at the Administrative Unit Level 2, we first scale the predicted fractions at the pixel level so that the predicted total maize fraction at Level 1 matches the FAO fraction. Then we plot the estimation results at Level 2 based on the scaled fractions.

**Fig 6:**
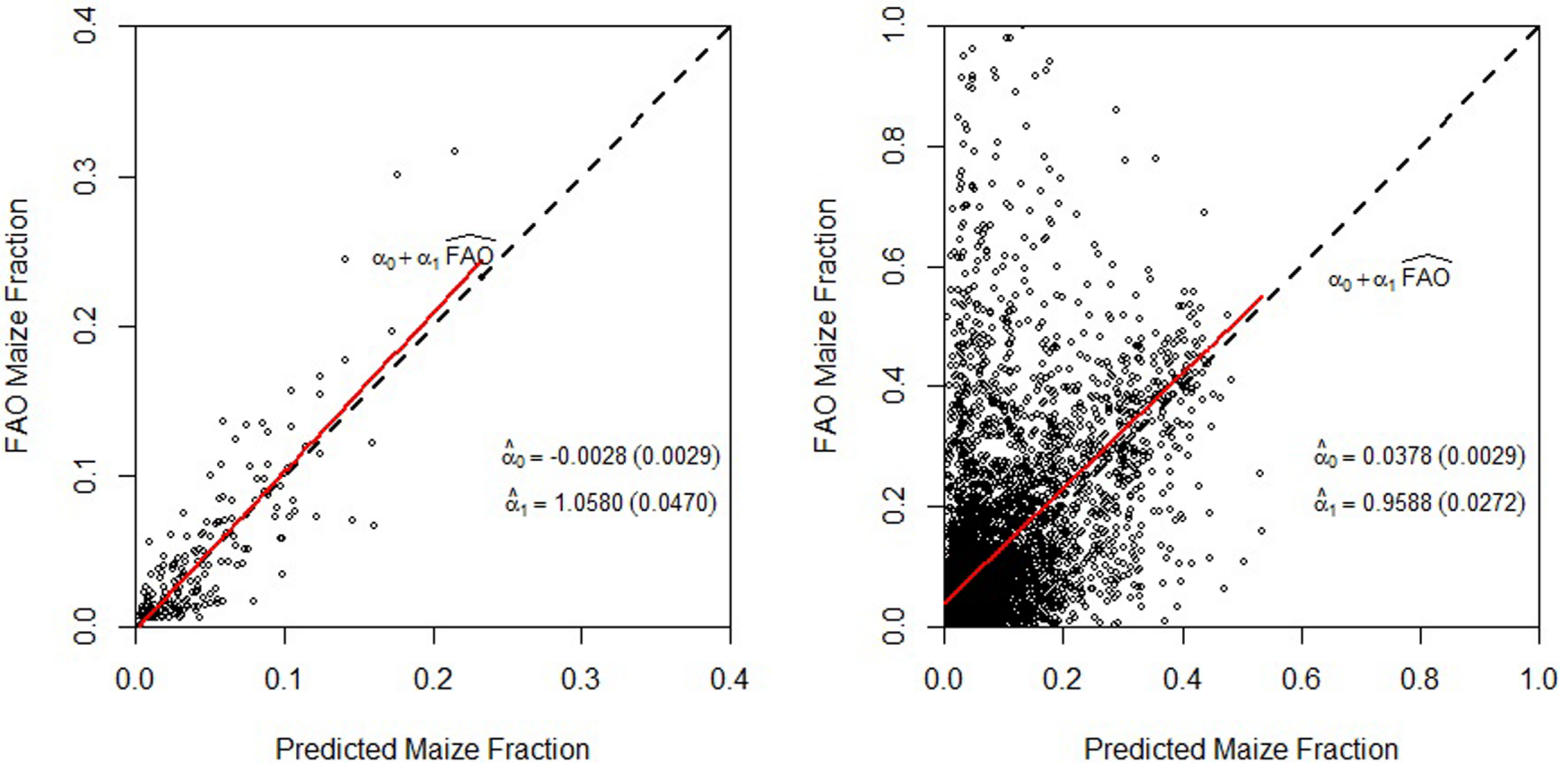
Comparison between the predicted maize area fraction versus observed FAO maize area fraction at Administrative Unit Levels 1 (left) and 2 (right).

Fig 6 graphically displays the prediction results at Level 1 and Level 2 for the single-crop maize model–the horizontal axis shows the model predicted maize fraction and the vertical axis shows the FAO maize fraction. The dashed diagonal line in each plot represents the 45 degree line; the closer the points are to the 45 degree line, the better the prediction. As is illustrated, the points generally cluster around the 45 degree line at both levels, indicating that the model generally predicts well. To more precisely measure the correlation between the predicted Level 1 fraction of maize and the observed FAO Level 1 maize fraction, we regress the predicted fraction on the observed FAO fraction. The regression line is shown by the solid line (in red) in each plot. In the case of ideal prediction, we expect that the intercept coefficient is equal to zero, and the slope coefficient is equal to one. At the Administrative Unit Level 1, the estimated intercept coefficient is -0.003, and is not statistically different from zero; the estimated slope coefficient is 1.06, and is not statistically different from 1. In addition, we compute the squared correlation between the predicted and FAO maize fraction as an R-squared, which is 0.72.

At Level 2, the intercept coefficient is 0.04, and is significantly different from zero; the slope parameter is 0.96, and is significantly different from one. The squared correlation between the predicted and FAO maize fraction is 0.15.

**Fig 7:**
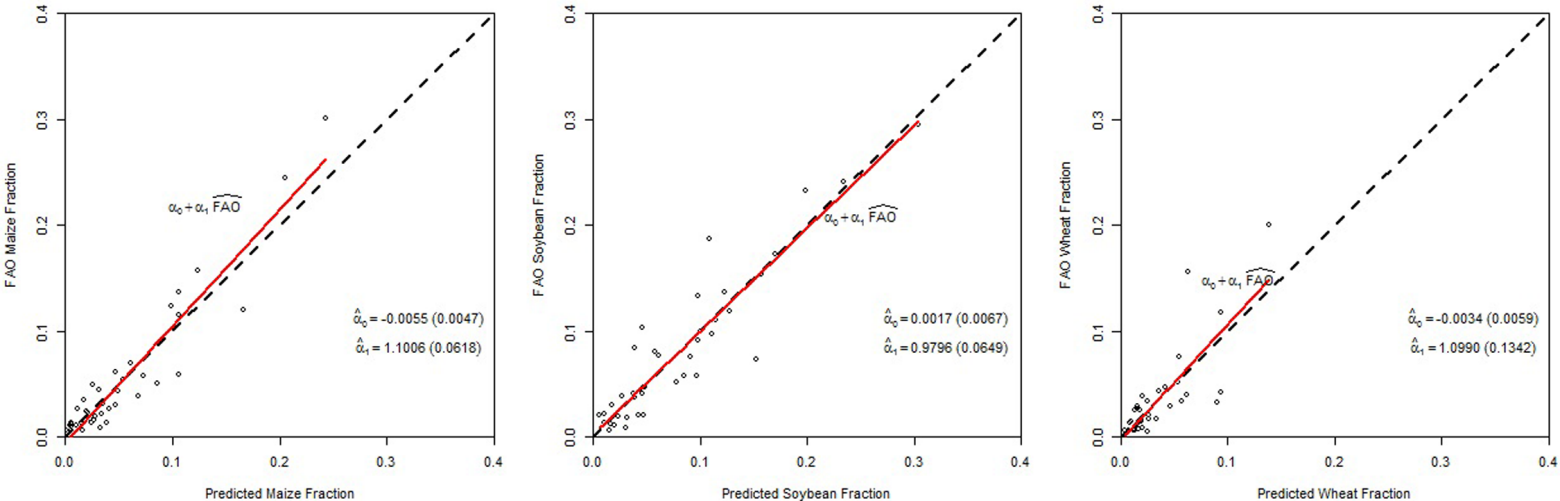
Comparison between the predicted area fraction versus the observed FAO area fraction for maize, soybeans, and wheat at Administrative Unit Level 1 for the multi-crop model.

**Fig 8:**
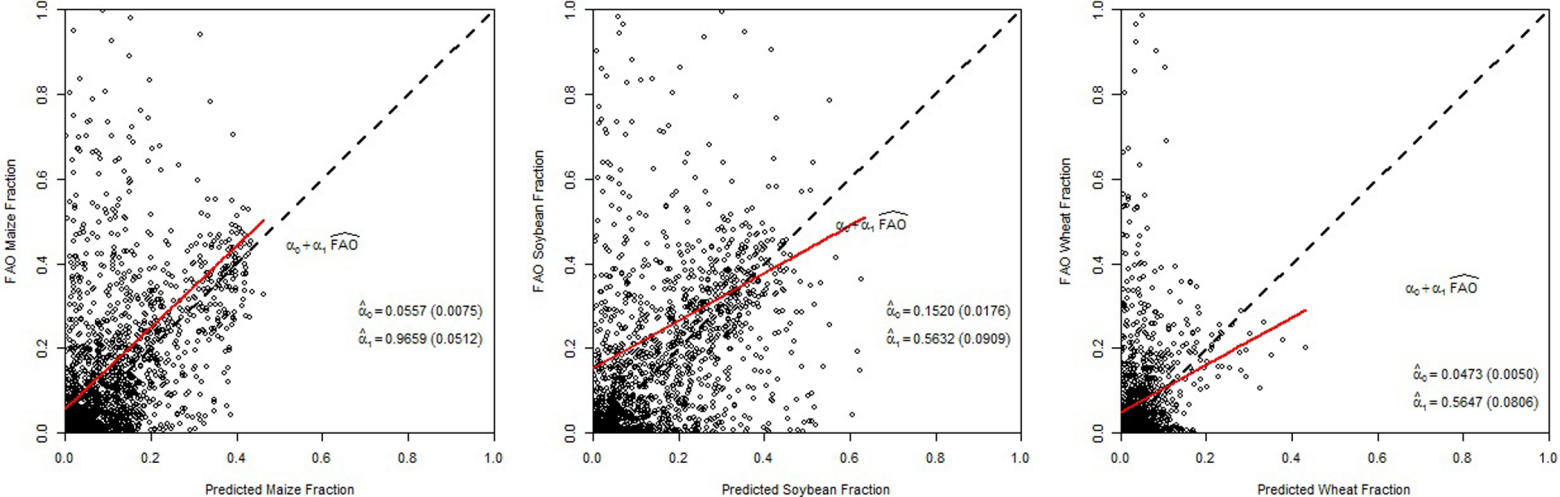
Comparison between the predicted area fraction versus the observed FAO area fraction comparison for maize, soybeans, and wheat at Administrative Unit Level 2 for the multi-crop model.

We report the results from similar validation exercises for the multi-crop model in Figs 7 and 8. The figures show that at both Administrative Unit levels the points are all close to the 45 degree line, which indicates that the multi-crop model predicts well by both in-sample and out-of-sample metrics. The squared correlation is 0.893 for maize, 0.857 for soybeans, and 0.638 for wheat at Level 1.

From these validation exercises, we find that at Level 1, for which we have crop harvested area data, the prediction results are closer to the FAO numbers than those at Level 2. However, considering our parsimonious set of land attribute variables, and that we do not have any crop specific variables, all the fractions are identified from the variation of crop shares at Level 1.

### Relative performance of the single and multi-crop models

To assess the relative performance of the single and multi-crop models, we calculate the root mean squared error (RMSE):

$$RMSE={\left[\frac{1}{J}\sum_{j=1}^{J}(predicted\;crop\;fraction_{j}-FAO\;crop\;fraction_{j}\right)}^{2}{]}^{\frac{1}{2}}.$$ (7)

Table 7 shows the RMSE values for the maize model (with 196 states) and the multi-crop model (with 40 states), both at Administrative Levels 1 and 2. For these samples, the multi-crop model predicts slightly better at Level 1 for the maize fraction compared to the maize model. It is possible that the relatively superior performance of the multi-crop model arises because it incorporates three crops; it is also possible that the differences in predictive performance arises because the samples are different. Both models predict relatively worse at Level 2 compared to each model’s own performance at Level 1; this is likely in part due to the fact that the Level 2 prediction is out-of-sample–this data is not directly used in the estimation.

**Table 7:** RMSE values for the maize model and the multi-crop model at Levels 1 and 2.

	Level 1	Level 2
*Maize Model*		
Maize	0.027	0.190
*Multi-crop Model*		
Maize	0.022	0.255
Soybeans	0.026	0.521
Wheat	0.024	0.152

We also consider the relative predictive performance of the single and multi-crop models using an identical sample of observations. Using the same 40 states, the RMSE values for the maize model are 0.040 at Level 1 and 0.259 at Level 2. Compared to the RMSE values for the multi-crop model in Table 7, the maize RMSE is slightly worse at both Level 1 and Level 2, which indicates that when the sample is the same, the performance of the multi-crop model is better.

### Validation against alternative data sources

To provide additional insight into the reliability of our cropland predictions, we validate our predictions against two additional sources. The first is the Monfreda et al. [8] predictions, given their widespread use in applied research. The second source is the USDA Cropland Data Layer (CDL), which is built on high resolution satellite images and agricultural surveys for several states in the United States and estimates crops growing in the field in June. For the available states, we take the CDL to be the benchmark (ground truth). For the year 2001 (the year of the United States data), the CDL has 30 by 30 meter pixel data for Illinois, Indiana, Iowa and North Dakota. To ensure comparability we aggregate the CDL pixels to match our pixel size and calculate the corresponding fractions. We compare the CDL crop shares to those from our maize model, multi-crop model (for maize), and the Monfreda et al. [8] predictions.

Table 8 shows the correlations at the pixel level for all four states across the four measures of predicted maize fractions. The correlations between different model estimates are relatively close for states where maize is the primary crop (Illinois, Indiana and Iowa). For North Dakota, other crops (wheat, soybeans, sunflowers, canola, and barley) all take higher percentages of cropland than maize (https://nassgeodata.gmu.edu/CropScape/); our models do not perform as well as the other models. We also check the performance of Monfreda et al. [8] for maize for all 196 states included in our estimation, at both Level 1 and Level 2. The Level 1 RMSE is 0.02, and the squared correlation is 0.88; the Level 2 RMSE is 0.20, and the squared correlation is 0.15. Considering that both the CDL data and Monfreda et al. [8] approach take into account a much larger number of crops, both are based on satellite imagery, and Monfreda et al. [8] also incorporate Level 2 if data is available, it is not surprising that their predictions are closer to each other. However, the results indicate that in general, we are able to achieve comparably good estimates based on a rather parsimonious set of dependent and independent variables, without relying heavily on satellite imagery.

**Table 8:** Correlations between different model estimated pixel specific maize fractions.

	Illinois	Indiana	Iowa	North Dakota
Maize Model vs. CDL	0.632	0.649	0.630	0.172
Multi-crop Model vs. CDL	0.605	0.601	NA	0.356
Maize Model vs. Monfreda et al.	0.759	0.672	0.610	0.289
Multi-crop Model vs. Monfreda et al.	0.752	0.628	NA	0.573
CDL vs. Monfreda et al.	0.779	0.846	0.784	0.728

Iowa is not included in our multi-crop model due to an insufficient land area for wheat.

### Illustrating the pixel-level predictions

We provide two illustrations of the pixel-level cropland predictions generated by our model: One for the studied 28 states in contiguous U.S., and the other for Hidalgo, Mexico. In each case, we compare predictions from a *naïve* approach against our predictions estimated using Level 1 FAO data. The *naïve* approach assumes that the fraction of maize in each pixel is equal to the Level 2 fraction of maize for the Level 2 region in which the pixel lies from USDA (in the U.S. case) or FAO (in the Hidalgo case). That is, a constant (pixel level) fraction within each Level 2 area. The second approach uses our maize model and the fitted, pixel-specific shares.

**Fig 9:**
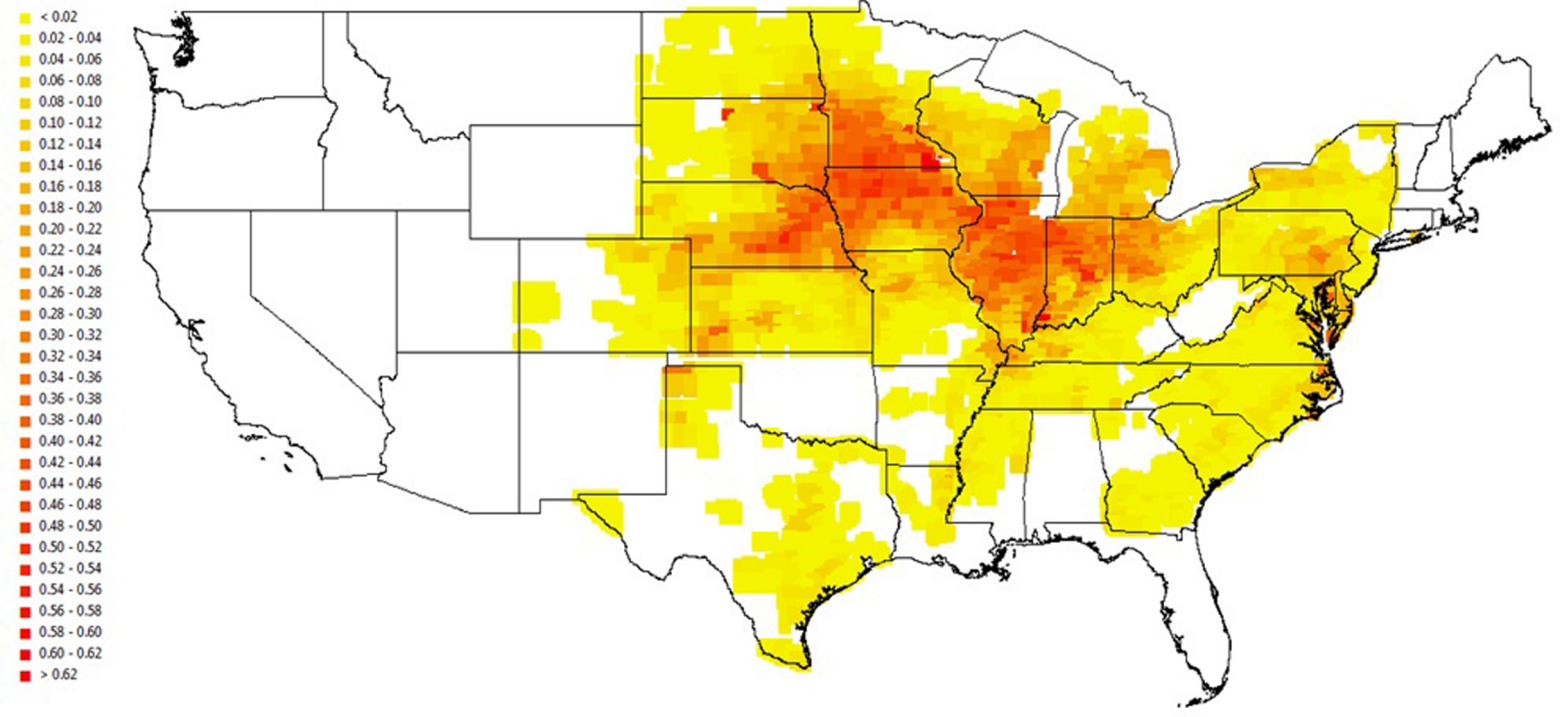
Pixel level maize fractions for the United States assuming constant FAO Level 2 shares for all pixels within each Level 2 area.

**Fig 10:**
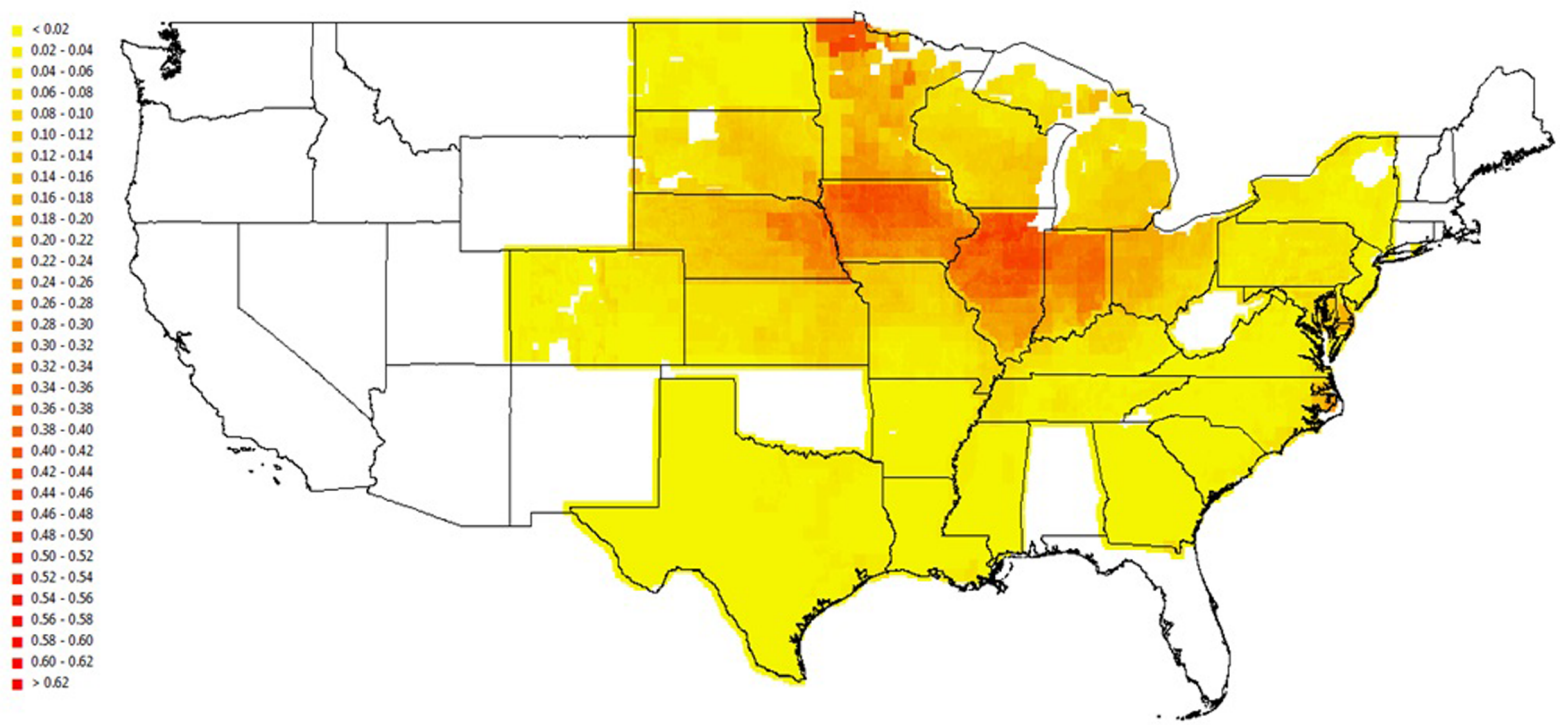
Pixel level maize fraction predictions for the United States using the estimated pixel-specific maize shares.

The first illustration is for the U.S. states. We generate two sets of downscaling predictions for harvested maize area in the United States–one with the average USDA Level 2 fraction applied to all pixels within a county where Level 2 data is available, and the other with our scaled predicted pixel level fractions. The USDA Level 2 data is highly reliable, allowing for a direct way of gauging the quality of our predictions. Fig 9 shows the USDA maize fraction plot at the pixel level assuming that all pixels within each Level 2 unit have the same maize fraction equal to the USDA Level 2 maize fraction for that unit. Fig 10 presents the pixel level predicted maize fraction plot scaled using the FAO Level 1 maize total harvested area. Colors from yellow to dark red indicate an increasing share of maize. The plots are alike for most areas, indicating that our model predicts well over a large geographic scale. The northwestern corner of Minnesota stands out as our model predicts a higher maize fraction than USDA. Given that USDA NASS data indicates the primary crops in that area are wheat and sugar beets, our over-prediction of maize is not surprising since our empirical model focuses on three major crops across a large geographical area only, rather than incorporating locally important crops such as sugar beets. But with the flexibility offered by our framework, a potential study is to build a more comprehensive model including more crops.

**Fig 11:**
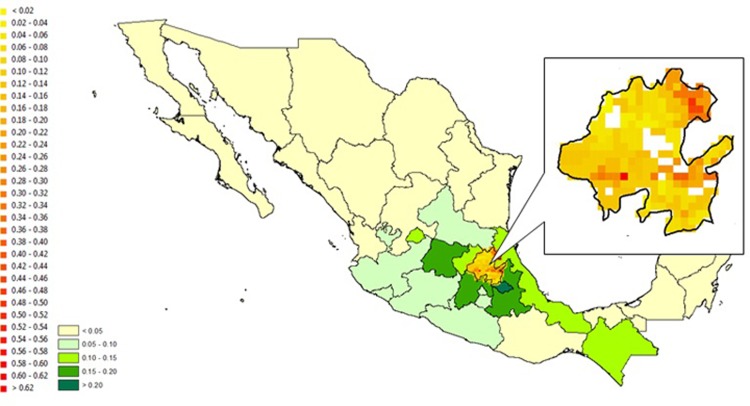
Pixel level maize fraction for Hidalgo, Mexico assuming constant FAO Level 2 shares for all pixels within each Level 2 area. *The Level 1 areas outside Hidalgo follow the color scheme of Fig 1.

**Fig 12:**
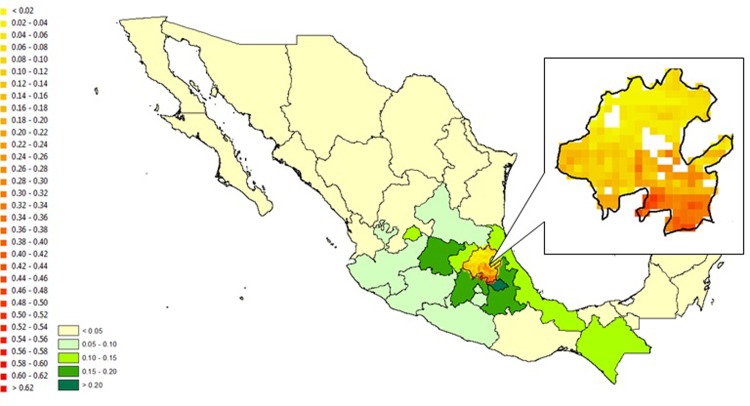
Pixel level maize fraction predictions for Hidalgo, Mexico using the estimated pixel-specific maize shares. *The Level 1 areas outside Hidalgo follow the color scheme of Fig 1.

Similarly, we compare estimations with the two approaches for Hidalgo, Mexico (shown in Figs 11 and 12). We pick Hidalgo as our example for two reasons. First, FAO Level 2 data is available for all 84 districts of Hidalgo, enabling us to fully compare the prediction results with FAO data. Second, there is relatively high variation in the fractions of maize within districts in Hidalgo, making it an interesting case for comparison.

Fig 11 shows that the constant fraction approach predicts that the pixels in the north-central part of Hidalgo have a lower fraction of maize, and the pixels in the north-eastern and part of the south-central regions have higher fractions of maize. The model results in Fig 12 are quite different: the model predicts that a larger number of pixels in the northern part of Hidalgo have a low fraction of maize, and a larger number of southern pixels have a higher fraction of maize. As a means of validation, we compare the FAO Agro-MAPS state level data with data collected from the Agrifood and Fisheries Information Service of Mexico (*Servicio de Información Agroalimentaria y Pesquera*, SIAP México). The correlation between FAO and SIAP data is 0.85, which provides reasonable assurance that the Level 1 FAO data for Mexico is reliable. However, district level harvested area data for maize is not available from SIAP, so we are not able to validate FAO district level data for Hidalgo. Nonetheless, further investigation confirms the credibility of our downscaled predictions: the south-eastern part of Hidalgo is home to the Valley of Tulancingo which is known for being one of the most fertile parts of the Valley of Mexico–a largely agricultural area. Further, the north-eastern part of Hidalgo is largely forest. Our model correctly identifies that the south-eastern part is the part of Hidalgo where most of the maize is grown; and there is less maize in the north-eastern area. These spatial patterns show that the *naïve* approach based on Level 2 FAO data and our estimation-based model can produce substantially different results. Anecdotal evidence supports that, at least in this case, our model seems to do a better job of predicting the location of agricultural activity than the FAO Level 2 data.

## Conclusions

We develop a statistical method for predicting pixel level cropland allocation across a (large) geographic area in which pixel-level measurements are not available. Specifically, we develop a fractional response model that combines measurements of pixel level land attributes with observable aggregate land use patterns to predict the share of cropland allocated to a certain crop at the pixel level. We formulate the likelihood function and demonstrate application to a single-crop model for maize and a multi-crop model for maize, soybeans, and wheat. We validate our estimates against other available spatially explicit cropland datasets and show that both the single-crop and multi-crop models at the Administrative Unit Levels 1 and 2 are reasonably precise in predicting cropland allocation at the pixel level.

Our statistical model and land allocation predictions provide applied scientists with measurements of land allocation at a pixel level, and with important advantages over previous measurements. First, the statistical framework is straightforward and transparent. This allows users of the model to gain a clear understanding of the relationship between the explanatory variables and the allocations. Second, while the model returns fractional estimates of cropland allocation in each pixel, the model also provides estimates of the marginal impacts of the variables on the pixel level allocations. These marginal impacts could be used in a variety of different contexts, to gain insight into how changes in biophysical attributes might impact cropland allocations. Third, the framework is flexible, and variables can be easily added if reliable data are available. For instance, if a variable such as travel time to markets is available, the model could be used to assess the impact of road infrastructure investments on cropping patterns. Irrigation is an important factor for cropland allocation in many regions across the world. Our search for global irrigation data only led us to data sources that do not appear to be generated independently from our dependent variable. If independently generated irrigation data becomes available over a broad geographic area, it would be straightforward to include it as an additional variable on the right hand side of our model. Fourth, the methods developed here can be used in any situation in which an aggregated variable needs to be downscaled to a finer resolution at which a vector of explanatory variables is available. Given pixel-level observations of our independent variables, the ideal situation would be to treat observations of the dependent variables at the most disaggregate level available–possibly at different levels in different areas. For example, U.S. data may be available at the county level, while reliable data for some other country may only be available at the state level. Finally, while we focus on North, Central and South America, our model can be readily applied to any continental or geographic area, such as Africa, where pixel level cropland allocation data is critically needed but not readily available.

## Supporting information

S1 Table Definition of variable measurement and data source. (DOCX)
