# Supplementary material for: Downscaling of national crop area statistics using drivers of cropland productivity measured at fine resolutions
Source: PLoS One. 2018 Oct 11;13(10):e0205152. doi: 10.1371/journal.pone.0205152 (PMC6181340; doi:10.1371/journal.pone.0205152)
Supplement: S1 Table — (DOCX) [file pone.0205152.s001.docx]

**Supporting information**

S1 Table. Definition of variable measurement and data source

| Name | Description | Source |
| --- | --- | --- |
| Temperature | Average monthly temperature in degrees Celsius over the period 1961-1990; For countries in the Northern Hemisphere the growing season is March through August, whereas the growing season for countries in the Southern Hemisphere is September through February. | New et al. (1999)  <http://www.sage.wisc.edu/atlas/index.php>, accessed May 25, 2015 |
| Precipitation | Average annual total precipitation in meters/year over the period 1961-1990. | New et al. (1999)  Same web link and access date as above |
| Elevation | Meters above sea level on a 5-minute resolution. | United States National Geophysical Data Center TerrainBase global model of terrain and bathymetry (1995)  Same web link and access date as above |
| Soil pH | Soil pH (0-14). | SoilData System, Global Soils Data Task, International Geosphere-Biosphere Program (IGBP-DIS) (1998)  Same web link and access date as above |
| Soil Carbon | Soil organic carbon density in kg per square meter, 0 to 1 meter depth. | Same source, web link and access date as above |
| Slope | Eight categories of median terrain slopes: 0-0.5%, 0.5-2%, 2-5%, 5-8%, 8-16%, 16-30%, 30-45% and > 45%. We use the median slopes of the IIASA/FAO slope categories as our slope variable values. | IIASA/FAO (2012) |
| -------------------------------------------------------------------------------------------------------------------------------------------------------- | | |
| Built-up Land | Combination of modeled built-up areas based on nighttime lights and observed built-up area based on IGBP land cover data. | <https://nelson.wisc.edu/sage/data-and-models/atlas/maps.php?datasetid=18&includerelatedlinks=1&dataset=18>, accessed Mar 04, 2016 |
| Protected Areas | Global raster data layer with a resolution of 5 arc-minutes. Each pixel is classified as protected area where agriculture should not be occurring, protected area where agriculture could be occurring, or non-protected area. | <http://www.fao.org/geonetwork/srv/en/main.home>, accessed Mar 04, 2015 |
| Total land area from Statoids | Total land area in an administrative unit. | <http://www.statoids.com/>, accessed Mar 05, 2016 |
| Harvested land area | Total areas of land harvested in maize, soybeans, and wheat at Administrative Unit Level 1. | <http://kids.fao.org/agromaps/>, retrieved Feb 20, 2015 |
| CIA World Factbook | Provides information on the government, geography, etc. for 267 world entities. | <https://www.cia.gov/library/publications/the-world-factbook/>, retrieved Feb 23, 2015 |
| GADM database | Spatial database on the location of the world’s administrative area. | <http://gadm.org/>, retrieved Feb 23, 2015 |
| USDA NASS Quick Stats | United States Department of Agriculture National Agricultural Statistics Service census and survey data. | <http://quickstats.nass.usda.gov/#528F56BC-9FFB-3942-B141-CA0EBDC414C9>, retrieved May 16, 2015 |
| USDA Cropland Data Layer | USDA National Agricultural Statistics Service Cropland Data Layer. | <https://nassgeodata.gmu.edu/CropScape/>, accessed Jan 19, 2016 |
